# Premature ovarian insufficiency: the context of long-term effects

**DOI:** 10.1007/s40618-016-0467-z

**Published:** 2016-04-18

**Authors:** A. Podfigurna-Stopa, A. Czyzyk, M. Grymowicz, R. Smolarczyk, K. Katulski, K. Czajkowski, B. Meczekalski

**Affiliations:** 1 Department of Gynecological Endocrinology, Poznan University of Medical Sciences, ul. Polna 33, Poznan, Poland; 2 Department of Gynecological Endocrinology, Warsaw Medical University, Warsaw, Poland; 3 II Department of Obstetrics and Gynaecology, Warsaw Medical University, Warsaw, Poland

**Keywords:** Premature ovarian insufficiency, Cardiovascular disease, Hypoestrogenism, Bone mineral density, Sexual dysfunction, Fertility

## Abstract

**Purpose:**

Premature ovarian insufficiency (POI) is defined as the cessation of the ovarian function before the age of 40 years. POI aetiology may be related to iatrogenic or endogenous factors and in many cases remains unclear. The aim of this review was to characterize the long-term consequences of POI.

**Methods:**

The available literature regarding the long-term consequences of POI from MEDLINE has been reviewed.

**Results:**

Lack of ovarian steroids synthesis has serious consequences for women’s health. The short-term effects are similar to spontaneous menopause and refer mainly to the climacteric syndrome. In a longer perspective, POI affects a variety of aspects. It obviously and drastically reduces the chances for spontaneous pregnancies. Oestrogen loss leads also to urogenital atrophy. The most common urogenital symptoms include vaginal dryness, vaginal irritation and itching. The urogenital atrophy and hypoestrogenism interferes also with sexual functioning. Patients with POI are threatened by a decrease in bone mineral density (BMD). POI women also experience psychological distress and some studies have shown an increased risk of neurodegenerating diseases. Overall, POI women have a shortened life expectancy, mainly due to cardiovascular disease. Some studies have reported a reduced risk of breast cancer in this group of patients.

**Conclusions:**

In conclusion there are several well-characterized health risks in POI women. With every patient, an individualized approach is required to properly recognize and prevent these risks.

## Introduction

Premature ovarian insufficiency (POI) is defined as an ovarian insufficiency before the age of 40 years [1]. It is characterized by a cessation of menstruation for at least 4 months associated with the elevation of serum follicle-stimulating hormone (FSH) concentration (FSH >40 IU/l) [2]. Development of hypergonadotropic hypogonadism before the age of 40 affects approximately 1–2 % of women [3].

Premature ovarian insufficiency can have an influence on premature morbidity and mortality [4]. It is related to profound hypoestrogenism, which has a deleterious effect on different systems in the female body [1]. For the first time, this condition was described by Fuller Albright in 1942 and defined as primary ovarian insufficiency [5].

POI can have a spontaneous or induced background. Spontaneous POI is referred to genetic, autoimmunological, inflammatory, enzyme deficiency, metabolic, or very often idiopathic causes [6, 7]. Induced POI occurs mainly due to oncological treatment such as surgery (bilateral oophorectomy), chemotherapy and radiotherapy [6]. The following factors have been associated with POI: heredity factors, ovarian surgery, pelvic surgery, chemotherapy and radiotherapy, exposure to toxicity and smoking, irregular menses in the history [8, 9].

The consequences of POI can be divided into short and long-term consequences.

**Fig. 1:**
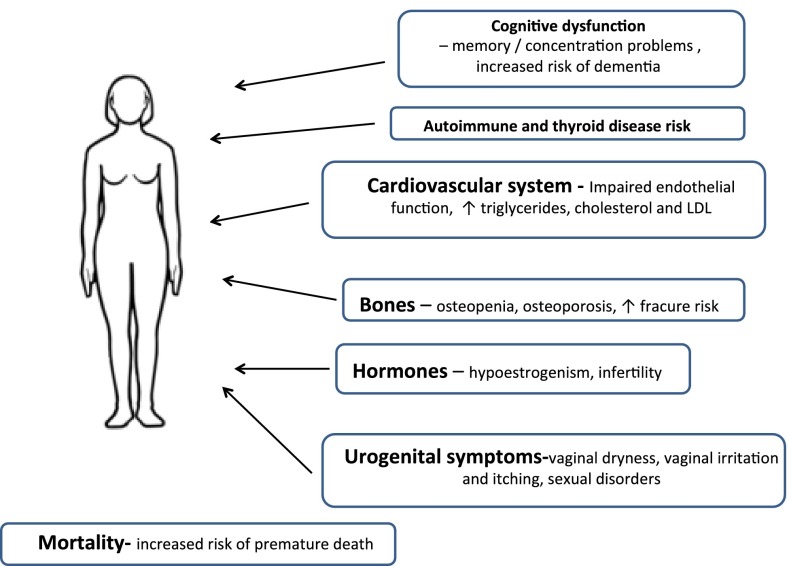
Long-term consequences of premature ovarian insufficiency–schematic summary

POI short-term consequences mostly result from prompt oestrogen deficiency. Short-term consequences may include vasomotor symptoms like hot flushes, night sweats, heart palpitations or headaches. The long-term health risks of POI are infertility, osteoporosis, cardiovascular and neurologic diseases and an increased risk of premature death [10]. Women with the diagnosis of POI also present psychological problems including irritability, forgetfulness, insomnia and poor concentration [6]. Moreover, women with POI may present vaginal atrophy, which is responsible very often for dyspareunia. The aim of this review was to summarize the available scientific data regarding POI long-term consequences (Fig. 1).

## Fertility, pregnancy, pregnancy outcome

Twenty five percent of POI cases has intermittent and unpredictable course, and the chance for spontaneous conception has been estimated to 4–10 % [11, 12]. The ovarian reserve of such women is assessed by an ultrasound count of antral follicles and serum anti-mullerian hormone (AMH), FSH, estradiol (E2), and inhibin B (InhB) measurements. A cohort of 358 patients with idiopathic POI followed up for almost 6 years was described by Bidet et al. [12]. The cumulative pregnancy course was 4.3 % at 48 months. Twenty-one spontaneous pregnancies resulted in 16 live births, one twin birth, four miscarriages and one elective abortion [12]. Two pregnancies were complicated by the occurrence of gestational diabetes mellitus, one by hypothyroidism, and one of the singleton pregnancies resulted in a preterm birth.

Little progress has been made to improve reproduction with patients’ own gametes. There are several studies evaluating the effectiveness of oestrogen, Gonadotropin-releasing hormone (GnRH) analogues, glucocorticoids or danazol pretreatment continued with ovarian stimulation with gonadotropins. Ovulation is achieved in approximately 20 % of POI patients. However, pregnancy rates for most of the strategies, assessed in systematic review by Robles et al. [13], were similar to the spontaneous pregnancy rates for these patients.

Oocyte donation is the only proven and recommended treatment for women with POI. The pregnancy rate after an oocyte donation cycle is around 40 % [14]. Cumulative pregnancy rates of oocyte donation treatment are very high and after four cycles reach 70–80 % [14]. Ameratunga et al. [15] reported following complications in 36 pregnancies after oocyte donation in POI patients: three cases of preterm labour, five gestational hypertensions, two gestational diabetes mellitus and one intrauterine growth retardation case. There were two twin gestations after a 2-embryo transfer.

There is an overall increase in cancer prevalence followed by an increase in long-term survival of the affected patients. Protection against iatrogenic POI caused by chemotherapy, radiation therapy or surgery assumes a high priority. Shielding or ovarian transposition during radiotherapy and fertility sparing surgery should be considered in young females undergoing cancer treatment. GnRH analogue given during chemotherapy significantly reduces the risk of POI in young cancer patients, but does not exhibit its protective effects in fertility [16, 17].

Cryopreservation of embryos and mature oocytes is the clinically established method, with pregnancy rates and livebirths reaching 25 % [18, 19].

All remaining options, such as retrieving immature oocytes aiming at maturing them later in vitro, freezing of gonadal tissue or novel methods using ovarian stem cells, are very promising but still considered experimental.

## Urogenital symptoms

Oestrogen loss leads to urogenital atrophy. The most common urogenital symptoms include vaginal dryness, vaginal irritation and itching [20]. The symptoms have been widely studied in women undergoing age-appropriate menopausal transition. However, there are few studies concerning the prevalence and treatment of genitourinary syndrome in POI patients. The frequency of most menopausal symptoms in the spontaneous POI group is generally lower than in medically induced premature menopause, with the exception of sexual function. It is worsened in more than half of affected patients in both groups [21].

The study evaluating the vaginal flora and the vaginal trophism of 36 women with POI under the age of 40 years, using hormonal therapy, found no significant differences regarding cytological aspects, pH measurements, vaginal microflora types and fungi infection comparing to age-matched women with normal gonadal function [22]. However, when sexual function was evaluated in both groups, women with POI showed worse sexual performance, with more pain and poorer lubrication than women in the control group [23]. These findings suggest that hormonal therapy in POI patients successfully reestablishes the epithelium cells, vaginal pH and microflora, but is not effective enough to improve complaints of lubrication and pain.

There are no studies reporting a higher occurrence of urinary incontinence among POI patients. Six POI women were studied urodynamically before and after the administration of oral and vaginal oestrogen. Oestrogen supplementation did not produce any significant changes in the functional or cystometric parameters. The authors concluded that oestrogen alone is of minimal significance in maintaining normal urinary tract physiology in the absence of ageing and other factors leading to urinary incontinence [24].

## Cancer risk

Cancer risk in POI patients is a complex issue. First, ovarian reserve depletion may occur as a result of cytotoxic cancer therapy in prepubertal or childbearing age patients and there is always concern about the cancer recurrence or subsequent neoplasms risk. Next, long-term hormonal treatment may carry additional cancer risk in subgroups of POI patients. Moreover, there are some epidemiological studies suggesting that the early cessation of ovarian function may be an independent cancer mortality risk factor.

There is no evidence that oestrogen replacement in spontaneous POI increases the risk of breast cancer in comparison with normally menstruating women [25]. Therefore, hormone therapy is recommended until the average age of the natural menopause and there is no need to start mammographic screening early [2]. It is, however, suggested that progestagens with the lower potential risk should be preferentially used [12]. Hormonal therapy with oestrogen and progestagens is regarded as contraindicated in breast cancer survivors [26]. Adolescent patients experiencing acute ovarian failure after cancer treatment will not go through puberty without oestrogen treatment [27]. However, in the subsets of childhood cancer survivors, such as Hodgkin disease survivors with the greater risk of breast cancer, heart disease and stroke, hormonal therapy implementation should be individualized [28].

A large study of middle-age and elderly Chinese women demonstrated that POI was inversely associated with the incidence of breast cancer [29]. This phenomenon is explained by the fact that the cumulative life-exposure of women with POI to endogenous circulating sex hormones is shorter than that of women with later menopause, leading to a reduced risk of breast cancer over a woman’s lifetime. Nevertheless, in the same study, POI has been associated with an increased risk of cancer mortality, and this increase was independent of hormonal replacement therapy [31].

## Cardiovascular disease

The main reason for shortened life expectancy in POI patients is cardiovascular disease and, therefore, some studies have addressed the issue of cardiovascular risk in this group of women. To date it has been shown that POI women present several risk factors for the development of cardiovascular disease: endothelial dysfunction, autonomic dysfunction, abnormal lipid profile, insulin action disturbances and metabolic syndrome.

Endothelial function, measured as the flow-mediated dilation of the brachial artery, has been shown to be significantly reduced in POI women. Similarly, the number of circulating endothelial progenitor cells number is diminished and correlated with a decreased serum estradiol concentration [30, 31]. The POI women present an increased carotid intima media thickness and left ventricular diastolic function [33]. Interestingly, the hormonal therapy of 6 months duration is able to improve the flow-mediated dilation by 2.4-fold, to the same levels as in healthy controls [32]. Goldemeier et al. [32] have also displayed normal endothelial-dependent vasodilation in POI women under hormonal therapy. Despite this, in the same study the authors showed impaired baroreflex sensitivity (3.9 ± 1.38 vs. 7.15 ± 3.62 ms/mmHg) and reduced heart rate variability (2310 ± 1173 vs. 3754 ± 1921 ms^2^) of POI women (*N* = 17) in comparison to healthy controls (*N* = 15) [34].

POI patients present abnormalities in lipid profile, but the results are conflicting regarding particular lipoproteins. As Knauff et al. [33] have reported, POI women (*N* = 90) show significantly higher TG levels (mean difference: 0.17 log mmol/L [95 % CI 0.06–0.29]) and lower HDL cholesterol levels in comparison to controls (*n* = 198) after correction for age, body mass index and smoking. This difference has not been confirmed in a smaller study (*N* = 47 POI vs. 60 controls) by Gulhan et al. [34]. However, this group revealed significantly higher TC and LDL levels in POI patients and a significant negative correlation between E2 and TC levels (*r* = −0.291, *P* = 0.047). Recently, Ates et al. [36] reported increased TC and HDL cholesterol in POI women (*N* = 59) vs. healthy controls (*N* = 59). The analysed population presented similar levels of glucose, insulin, HOMA-IR, low-density lipoprotein cholesterol (LDL-C), and triglyceride as the controls, but the incidence of metabolic syndrome was significantly increased [35]. In contrast, other authors detected increased serum glucose, insulin and the homeostasis model of assessment-insulin resistance (HOMA-IR) in POI women (*N* = 43) vs. controls (*N* = 33) [36].

Even though there are conflicting data regarding lipid profile and insulin resistance indices, the overall cardiovascular risk in POI women seems to be significantly increased, as the mortality causes analyses in this group has shown (above). Especially the risk of mortality from ischaemic heart disease is approximately 80 % increased in the POI women group compared to women with menopause at 49–55 years [37].

## Bone mineral density

An association between oestrogen deficiency in the postmenopausal period and osteoporosis has been clearly established (North American Menopause Society) [38]. Albright et al. [39] publications are the first to demonstrate the relationship between oestrogen deficiency, menopause and an increased incidence of fractures in women [41].

However, in young women hypoestrogenism and hypoandrogenemia have a deleterious effect on peak bone mass (PBM) formation and bone mineral density (BMD) status [40]. Lana et al. [41] found that serum FSH concentrations, but not oestradiol, are positively associated with bone mass loss in skeletal regions (both the spinal column and femoral neck) in patients with spontaneous POI.

Numerous studies have revealed a significant decrease of BMD in POI patients. Uygur et al. [42] found that both the femoral neck bone and spinal bone BMD were significantly lower in POI patients than measurements of the control group.

A large study conducted by Popat [43] including 442 cases confirmed that POI patients have a lower BMD compared to regularly menstruating women. According to a study by Nelson et al. [44] 67 % of patients with POI have osteopenia. Leite-Silva et al. [45] studied 50 women with POI and found a decrease in the lumbar spine and femoral BMD. The lumbar part of the spine was the most affected by the BMD decrease. They reported that age generally, age of POI and reproductive age were factors associated with the BMD of the lumbar spine.

Total body BMD clearly corresponds to the duration of ovarian function in POI patients [46].

There are limited data regarding fracture risk in POI patients. Clinical studies that compared women experiencing menopause at normal age to women who had premature menopause reported relative risks for fracture of approximately 1.5 in women with premature menopause [47].

## Autoimmune and thyroid disease risk

It is estimated that 4–30 % of POI cases are due to autoimmune aetiology. Therefore, the autoimmune diseases risk is increased in this group [48]. According to available data, the incidence of autoimmune hypothyroidism, adrenal insufficiency, diabetes type 1, hypoparathyroidism and pernicious anaemia is increased [49].

Autoimmune thyroiditis seems to be the most common autoimmune disease in the POI population. Up to 24 % of POI women were reported positive for thyroid peroxidase autoantibodies [50]. The overt hypothyroidism is present in about 8–20 % of POI individuals [51, 52]. 21-hydroxylase adrenal autoantibodies have been reported in 3.2 % of POI women. Similarly, approximately 2–3 % of women will have asymptomatic autoimmune adrenal insufficiency [52, 53]. According to another report, women with adrenal autoimmunity detected by the presence of autoantibodies have a 50 % risk of developing adrenal insufficiency [53].

The incidence of type 1 diabetes in POI, according to available reports, is 2.5 % [54]. The reports about the association of POI with other autoimmune diseases are anecdotal; therefore, these disorders are relatively uncommon.

It has been also shown that non-autoimmune POI can be either linked to increased risk of developing autoimmune disease. In Nurses Health Study, early menopause (including surgical) early menopause (at age <45 years) has been related to augmented risk of systemic lupus erythematosus and rheumatoid arthritis [54]. The pathophysiological mechanisms related to these phenomena seem to be complex. Probably the immunomodulatory effects of estradiol (especially on T helper cells) are of importance, but this issue requires further evaluation [55].

## Cognitive dysfunction

Work by several groups has provided evidence that early menopause can be associated with neurological dysfunction and an increased risk of dementia. The data suggest an increased risk of neurological disorder where POI is due to premature menopause or induced from surgery. This increased risk appears to be most apparent in the domains of global cognitive and verbal memory tests. Where POI is caused by genetic disorder, observed cognitive deficiencies may be more likely to have a genetic basis rather than being due to the effects of sex steroids on the brain. Findings related to the loss of cognitive function after chemotherapy or GnRH analogues treatments are mixed.

Nappi et al. [56] investigated attentive and verbal memory performances in physiological and surgical menopause, drawing attention to the impact of age at menopause. Surgical menopause affects short-term verbal memory more than physiological menopause.

A recent publication of Bove et al. [57] determined the association between age at surgical menopause and both cognitive decline and Alzheimer’s disease in two longitudinal cohorts. The researchers found that early age at surgical menopause was associated with cognitive decline and the incidence of Alzheimer’s disease.

In a longitudinal study, Farrag et al. [58] investigated the effect of oestrogen deficiency on cognitive function in surgically menopausal women (*n* = 35). Rapid decline in the oestrogen level following surgical menopause was associated with a deleterious effect on cognitive function.

Another study from the Mayo Clinic Cohort Study of Oophorectomy and Ageing, investigated women who had both ovaries removed before reaching natural menopause. The authors found that examined women experienced a long-term increased risk of Parkinsonism, cognitive impairment or dementia, depressive and anxiety symptoms [59].

Phung et al. [60] revealed that premenopausal bilateral oophorectomy is associated with a higher risk of dementia, suggesting a dose effect of premature oestrogen deficiency on dementia. The age-dependent effect suggests that the younger brain is more vulnerable to oestrogen deficiency.

## Mood and sexual disorders

The psychosocial aspects of POI are most often ignored in the context of the diagnosis. The decrease in the mood can be caused not only by concerns about their own health, but also reproductive problems that occur in younger women who want to have children. Being diagnosed with POI can be an unexpected and upsetting diagnosis. Women with POI experience significant psychologic disturbances, such as high levels of depression and low levels of self-esteem, with negative effects on sexuality [61]. The diagnosis of POI can be an extremely devastating life experience and patients often express anger, depression, anxiety, loss and sadness. Women after being informed of POI diagnosis can be shocked and confused. These words are describing their emotional trauma. Some of POI patients report a range of emotions and providers should offer support regarding the patient’s altered self-image, sexual dysfunction and neurocognitive decline.

Schmitdt et al. [62] evaluated depression in women with spontaneous 46, XX primary ovarian insufficiency. The researchers compared prevalence of depressive symptoms observed in women with primary ovarian insufficiency (POI) with women in whom the menopause is normally timed. They studied 174 women with spontaneous 46, XX POI and 100 women with Turner syndrome. POI proved to be associated with an increased lifetime risk for major depression. The scientists stressed that more attention should be paid to the presence of depression in POI. Moreover, the onset of depression frequently occured after signs of altered ovarian function but before the diagnosis of POI.

Fang et al. [63] prospectively compared quality of life (QOL) in 75 women at increased risk of ovarian cancer who are undergoing risk-reducing salpingo-oophorectomy. Women who underwent surgery revealed poorer physical functioning, greater pain, less vitality, decreased social functioning, greater discomfort and less satisfaction with sexual activities at 1 month compared to controls. However, most QOL deficits observed in the surgical group were no longer apparent by the 6-month assessment.

A cross-sectional study comparing sexual function of 58 POI patients with eumenorrheic women revealed that POI patients experienced worse sexual function in relation to satisfaction, lubrication, orgasm, pain and arousal. However, there were no differences between the two groups with respect to desire [64]. Similar results showed another study including 81 POI patients complaining of less sexual arousal, reduced lubrication and increased genital pain, without a disturbed feeling of desire comparing to healthy controls [65]. The POI patients in this study had decreased serum androgen serum levels in comparison to the control group. Detailed analysis of the hormonal profiles of the study group revealed the weak influence of androgen on sexual functioning. Higher total testosterone levels were associated with an increased frequency of desire, and higher androstenedione levels were associated with an elevated frequency of sexual contact [67].

Dennerstein et al. [66] determined the prevalence of hypoactive sexual desire disorder (HSDD) among women who have undergone surgical menopause with that of premenopausal or naturally menopausal women. The surgical menopausal women are at an increased risk for HSDD. HSDD is associated with diminished sexual and partner relationship satisfaction and negative emotional states.

## Mortality

Overall mortality is increased in women with POI. According to data from a cohort of 19,731 women from Norway found that the menopause before 40 years old was linked to an increased mortality rate of 1.06 (CI 0.99–1.14) in comparison to women who had menopause at the age 50–52 [67]. Also early menopause has been shown to reduce the life expectancy. Data from a prospective cohort study of 68,154 US adult women showed that all-cause mortality rates were higher among women who reported that their menopause occurred at age 40–44 years compared with women who reported that their menopause occurred at age 50–54 years [rate ratio (RR) = 1.04, 95 % confidence interval (CI) 1.00, 1.08] [68].

The increased risk of all-cause mortality is mainly dependent on higher mortality rates from coronary heart disease (RR = 1.09, 95 % CI 1.00, 1.18), respiratory disease (RR = 1.19, 95 % CI 1.02, 1.39), genitourinary disease (RR = 1.39, 95 % CI 1.07, 1.82) and external causes (RR = 1.56, 95 % CI 1.21, 2.02) [69].

## Summary: clinical implications

POI patients have an increased risk of certain morbidity as well as mortality, as shown above. Recently, the European Society of Human Reproduction published management guidelines for POI [69]. According to expert opinion women with POI should be advised on how to reduce cardiovascular risk factors by not smoking, by taking regular exercise and maintaining a healthy weight to reduce the risk of premature death. The cardiovascular evaluation should consist of monitoring annually blood pressure, weight and smoking status. As we have stated above, the chance for spontaneous pregnancy is very low; therefore, oocyte donation is the best option for fertility. The risk of BMD decrease and osteoporosis requires proper management, i.e. lifestyle interventions, sufficient intake of calcium and vitamin D and hormonal replacement therapy. All the patients diagnosed with POI should be evaluated for BMD (densitometry). Due to the risk of psychological problems, patients should be allowed to obtain psychological help. Sexual dysfunction should be managed by proper counselling, oestrogen replacement and androgen supplementation in chosen cases. Vaginal atrophy may by improved by the use of topical oestrogens and lubricants. According to expert opinion, the neurological well-being should be supported by healthy lifestyle interventions and oestrogen replacement.
